# Bar adsorptive microextraction and liquid chromatography-diode array detection of synthetic cannabinoids in oral fluid

**DOI:** 10.1007/s00216-024-05517-0

**Published:** 2024-09-11

**Authors:** Samir M. Ahmad, Nuno R. Neng, Cláudio R. Queirós, Helena Gaspar, José Manuel F. Nogueira

**Affiliations:** 1 Centro de Química Estrutural, Institute of Molecular Sciences, Departamento de Química e Bioquímica, Faculdade de Ciências, Universidade de Lisboa, Campo Grande, 1749-016 Lisboa, Portugal; 2 BioISI - Biosystems & Integrative Sciences Institute, Faculdade de Ciências, Universidade de Lisboa, Campo Grande, 1749-016 Lisboa, Portugal

**Keywords:** Synthetic cannabinoids, Drug control, Bar adsorptive microextraction, HPLC-DAD, Oral fluids

## Abstract

In recent years, synthetic cannabinoids (SCs) have become a major public health issue. For this reason, there is a need for innovative analytical methods that allow its monitoring in biological matrices. In this work, we propose a novel methodology to screen eight SCs (AM-694, cumyl-5F-PINACA, MAM-2201, 5F-UR-144, JWH-018, JWH-122, UR-144 and APINACA) in oral fluids. A bar adsorptive microextraction method followed by microliquid desorption combined with high-performance liquid chromatography with diode array detection (BAµE-µLD/HPLC-DAD) was developed to monitor the target SCs. The main factors affecting the BAµE technology were fully optimized for oral fluid analysis. Under optimized experimental conditions, the proposed methodology showed good linear dynamic ranges from 20.0 to 2000.0 µg L^−1^ (*r*^2^ > 0.99, relative residuals < 15%), limits of detection between 2.0 and 5.0 µg L^−1^ and suitable average recovery yields (87.9–100.5%) for the eight studied SCs. The intra- and interday accuracies (bias ≤ ± 14.7%) and precisions (RSD ≤ 14.9%) were also evaluated at three spiking levels. The validated methodology was then assayed to oral fluid samples collected from several volunteers. The proposed analytical approach showed remarkable performance and could be an effective alternative for routine monitoring of the target compounds in oral fluid.

**Graphical Abstract:**

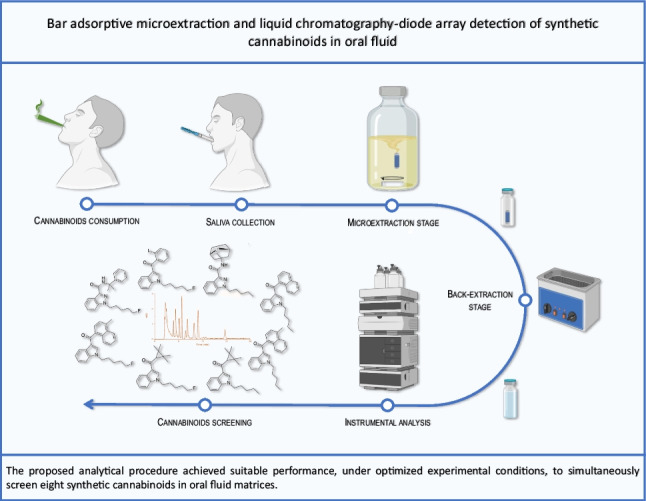

**Supplementary Information:**

The online version contains supplementary material available at 10.1007/s00216-024-05517-0.

## Introduction

Synthetic cannabinoids (SCs) are defined as all substances that intent to mimic the effects of (-)-*trans*-Δ^9^-tetrahydrocannabinol (THC), the main active ingredient of cannabis. These compounds interact with the endocannabinoid system, in particular with the CB1 and CB2 receptors [1]. This mechanism is involved in several physiological processes, such as appetite and sleep regulation, the sensation of pain, cognition, among many others. In comparison to THC, some SCs have four to five times greater affinity to these receptors [2]. Consequently, several toxicity symptoms have been reported. These include numbness, anxiety, high blood pressure, paranoia, tachycardia, irritability, hallucination, seizures, sleepiness, slurred speech and, ultimately, even death [3]. The use of specific SCs were linked with 21 deaths in Hungary in 2020 [4]. Moreover, 15 SCs were reported for the first time in 2021, which add to a total of 224 that have been detected in Europe since 2008 [5]. For all these reasons, new analytical methodologies must be implemented that allow effective monitoring of SCs in different types of biological matrices, particularly using non-invasive methods, such as oral fluid sampling. Other advantages of using this biological matrix are its rapid sample collection and the absence of health risks; it still represents the free fraction of a drug and can hardly be adulterated [6]. So far, several analytical approaches have been suggested to control SCs in oral fluids [7–13], usually including a sample preparation step, i.e. liquid-liquid extraction (LLE) or solid-phase extraction (SPE) prior to liquid chromatography coupled to mass spectrometry (LC-MS) or using tandem systems (LC-MS/MS) [14]. Recently, our group introduced alternative passive-based microextraction devices, bar adsorptive microextraction (BAμE), which operate under the floating sampling mode and use nanostructured and polymeric materials as sorbent phases. This technique has shown to be a successfully alternative for monitoring several types of priority and emerging organic compounds having distinct physicochemical characteristics in aqueous media [15, 16], including drugs of abuse [17–22].

**Fig. 1:**
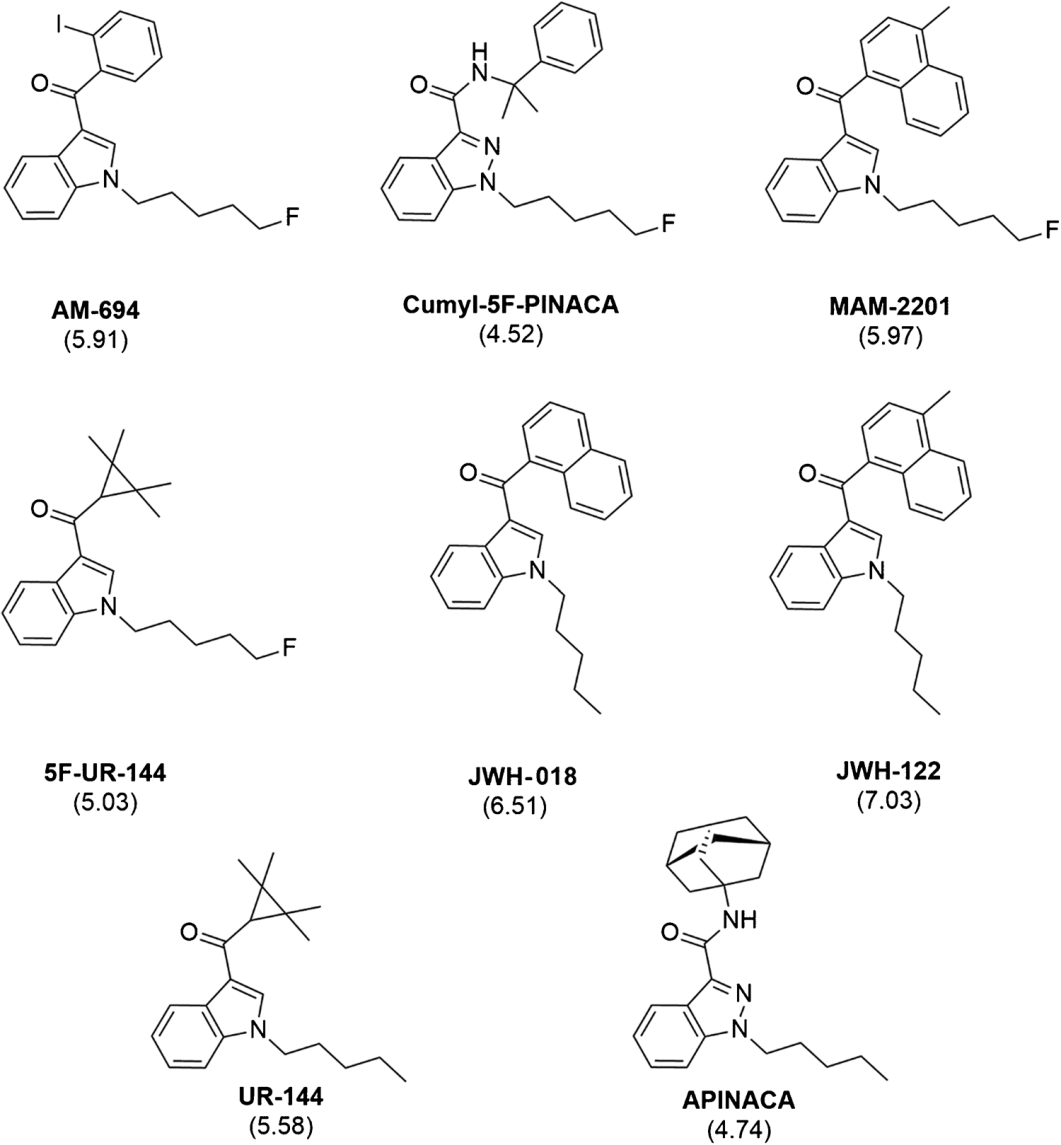
Chemical structures of the eight SCs under study. In parenthesis is presented the respective log *P* values [22]

In this contribution, we propose the development, optimization, validation and application of BAμE in combination with high-performance liquid chromatography with diode array detection (BAµE-µLD/HPLC-DAD), to monitor eight SCs in oral fluids; AM-694 [(1-(5-fluoropentyl)indol-3-yl)-(2-iodophenyl)methanone, cumyl-5F-PINACA [1-(5-fluopentyl)-*N*-(2-phenylpropan-2-yl)indazole-3-carboxamide], MAM-2201 [(1-(5-fluoropentyl)-1H-indol-3-yl)(4-methyl-1-naphthyl)methanone], 5F-UR-144 [(1-(5-fluoropentyl)-indol-3-yl)(2,2,3,3-tetramethylcyclopropyl)methanone], JWH-018 [naphthalen-1-yl-(1-pentylindol-3-yl)methanone], JWH-122 [(4-methylnaphthalen-1-yl)-(1-pentylindol-3-yl)methanone]], UR-144 [(1-pentylindol-3-yl)-(2,2,3,3-tetramethylcyclopropyl)methanone] and APINACA [N-(1-adamantyl)-1-pentylindazole-3-carboxamide]. Figure 1 depicts the chemical structures of the target SCs as well as their respective log *P* values [23].

## Materials and methods

### Chemicals, materials, samples and instruments

The HPLC-grade methanol (MeOH, 99.8 %), acetonitrile (ACN, 99.8 %) and ethanol absolute anhydrous (EtOH, 99.9 %) were purchased from Carlo Erba (Italy). Sodium chloride (NaCl, 99.9 %), sodium hydroxide (NaOH, 98.0 %) and propan-1-ol (1-PrOH, 99.8 %) were obtained from AnalaR BDH Chemicals (UK). Propan-2-ol (2-PrOH, 99.9 %) was obtained from Fisher Scientific (UK). The hydrochloric acid (37.0 %) and sodium carbonate (99.5%) were purchased from Riedel-de Haën (Germany). Ultra-pure water was obtained from Milli-Q water purification systems (USA). The sorbent phases used for the preparation of the microextraction devices were Strata®-X (N-vinylpyrrolidone and divinylbenzene copolymer; 33 μm particle size, 800 m^2^ g^−1^ surface area, pH 1–14 stability) and Ciano (silica and ciano copolymer, particle size 55 μm, surface area 500 m^2^ g^−1^, pH 1–14 stability) from Phenomenex, USA; LiChrolut® EN (ethylvinylbenzene and divinylbenzene copolymer; particle size 40–120 μm, surface area 1200 m^2^ g^−1^, pH 1–13 stability) from Merck Millipore, Germany; HLB (N-vinylpyrrolidone and divinylbenzene copolymer, 30–60 μm particle size, 810 m^2^ g^−1^ surface area, pH 0–14 stability) from Waters, USA); and ENVI™-18 (octadecyl silica polymer, 45 μm particle size, 475 m^2^ g^−1^ surface area) from Supelco, PA, USA. Seven out of the SCs selected as model compounds used in this work were previously isolated and characterized from products supplied by the Scientific Police from Portuguese Criminal Police (Lisbon, Portugal, which also provided a standard of cumyl-5F-PINACA) [24]. All compounds were characterized by nuclear magnetic resonance (NMR). Briefly, an aliquot of each SC (10 to 15 mg) was dissolved in CDCl_3_ for NMR structural analysis. ^13^C NMR (100.6 MHz) and ^1^H NMR (400.1 MHz) were recorded on a Bruker Avance spectrometer. The chemical shifts were expressed as *ẟ* values and referenced to the residual solvent peak (CDCl_3_, *ẟ*H=2.50, *ẟ*C=39.5). Units of Hertz (Hz) were used for reporting the coupling constants. 1D (^1^H, ^13^C APT) and 2D (COSY, HMBC and HSQC) NMR experiments showed unequivocal assignments of all proton and carbon signals. All NMR results obtained were similar to previous literature reported data [24–29].

Blank assays were performed in oral fluid samples provided by the academic community (volunteers), obtained in 2018 from 10 individuals (5 female and 5 male) by passive drooling into polypropylene cryovials [6]. It was requested that the subjects did not eat, drink or smoke for at least 10 min before the samples collection. Additionally, the volunteers guaranteed that no drugs of abuse were consumed for at least 24 h prior to sampling. Assays performed on real oral fluid samples were also obtained from the academic community (volunteers) following the same procedure as above, but without the restrictions used for the blank assays. For non-disclosure purposes, the analysis was performed without any information from the donor. After collection, the vials were kept at −20 °C, to avoid drug losses, as previously demonstrated [30]. When possible, the samples were analysed on the same day. The study was conducted according to the guidelines of the Declaration of Helsinki and approved by the Institutional Review Board of the Faculdade de Ciências da Universidade de Lisboa (‘Comissão de Ética para Recolha e Proteção de Dados’) nr 02/2020.

The HPLC-DAD analyses were carried using similar equipment and strategy as already described in the literature [20] with a few modifications described below. In this specific case, we employed a Kinetex C18 column, 150.0 × 4.6 mm, 2.6 µm particle size (Phenomenex, Torrance, USA). The samples were analysed using a mobile phase consisting of an isocratic mixture of water and ACN (20/80 %, v/v). The detector was set at 302 nm, and the injection volume was 20 µL.

### Experimental setup

#### Preparation of the BAµE devices

The BAµE devices were prepared within the laboratory in accordance with existing research literature [31, 32]. Each bar, measuring 7.5 mm in length and 3 mm in diameter, underwent a meticulous process of coating with powdered polymeric phase, utilizing a suitable adhesive film. Subsequently, they were securely stored at room temperature in a hermetically sealed glass flask. The microextraction devices were intentionally designed for single use, owing to their affordability, ease of preparation, and to mitigate any potential carry-over effects. Prior to utilization, the BAµE devices were subjected to a thorough cleaning regimen involving the use of MeOH and ultra-pure water.

#### BAµE-µLD method development

The optimization experiments were performed in glass flasks having 10 mL of ultra-pure water spiked with the target SC to get a concentration 20.0 µg L^−1^. Afterwards, a BAµE device coated with a specific sorbent material and a stir bar were introduced in the flask. The assays were performed at room temperature (25 ºC in a multipoint agitation plate (Variomag H+P Labortechnik Multipoint 15, Oberschleissheim, Germany). To optimize the microextraction method, several parameters affecting its performance were evaluated. This included coating selectivity, stirring rate (750, 1000 and 1250 rpm), equilibrium time (1, 2, 3, 4 and 16 h), organic modifier (MeOH; 5, 10 and 15 %, v/v), matrix pH (2.0, 5.5, 8.0 and 11.0), and ionic strength (NaCl; 5, 10 and 15 %, w/v). After the microextraction process, the BAµE devices were taken out of the flasks with clean tweezes and put into glass vial inserts containing 100 μL of the back-extraction solvent. This was followed by ultrasonic treatment (Branson 3510, Zurich, Switzerland) at room temperature. For microliquid desorption (µLD) stage, several solvents were tested to achieve the best results. These included ACN, MeOH, EtOH, 1-PrOH and 2-PrOH, at several periods of sonication time (5, 15, 30, 45 and 60 min). Afterwards, the devices were removed from the inserts with clean tweezes, and the vials were sealed and placed on the autosampler for HPLC-DAD analysis. In supplementary material S1, we provide a simplified schematic diagram of the proposed experimental procedure (Figure S1).

### BAµE-µLD/HPLC-DAD validation assays

For the validation assays, oral fluid samples were subjected to homogenization by vortexing for 30 s, followed by the addition of ACN for protein precipitation [33] at a volumetric ratio of 1:2 (ACN/sample). Subsequently, the samples were vortexed again for 30 s and then centrifuged at 4000 rpm for 10 min using a Hermle Z 300 centrifuge (Germany). Next, 750 µL of the resulting supernatant was transferred to a sampling flask, and 8.5 mL of ultra-pure water, and 1 mL of MeOH was added. This was followed by the implementation of the optimized methodology using BAµE-µLD/HPLC-DAD.

Several parameters were evaluated for the purpose of validation, including sensitivity, selectivity, linearity, precision, accuracy and recovery yields, as described in the relevant literature [33–35]. Unless otherwise specified, all validation assays were conducted in triplicate.

#### Linearity, sensitivity and selectivity

The linearity was checked by spiking oral fluid samples (before ACN addition) with a standard mixture to get a final concentration in between 20.0 and 2000.0 µg L^−1^ (ten points), followed by using the optimized methodology. The results enabled the production of a least-squared regression plot. The acceptance criteria were that all the points in the curve should have a relative deviation ≤ 15.0 % from its nominal concentration (relative residuals, RR) and that the determination coefficient (*r*^2^) should be ≥ 0.99. The limits of detection (LODs) and quantification (LOQs) estimated with a signal-to-noise ratio (S/N) of 3/1 and 10/1, respectively, using the same approach described above. The selectivity was assessed by evaluating the interferences that could occur due to endogenous materials in the oral fluid samples. This was accomplished by applying the optimized methodology to ten non-spiked samples (ten drug-free volunteers) and by checking the resulting chromatograms for any interfering compounds, especially at the retention times of each compound.

#### Accuracy and precision

Method intraday (*n* = 6, 5 consecutive days) and interday (*n* = 6) accuracies and precisions were assessed by spiking oral fluid samples (before ACN addition) to get a final concentration of 50.0, 300.0 and 1000.0 µg L^−1^. Accuracy was calculated as bias:

$$Bias=\frac{E-T}{T} \times 100$$

where *E* is the experimentally determined concentration and *T* is the expected concentration for spiked samples. The interday and intraday precisions were calculated as relative standard deviation (RSD) from the interday and intraday accuracy assays, respectively. The acceptance criteria for accuracy and precision were that bias and RSD should be ≤ 15.0 %, respectively.

#### Recovery yields

The recovery assays were performed as described in the literature [34–37]. In brief, two distinct sets of samples, labelled A and B, were prepared at varying concentrations of 50.0 and 300.0 µg L^−1^. Set A, comprising six samples, represented a pure standard mixture. Set B, also consisting of six samples, was created by spiking oral fluid samples obtained from different drug-free volunteers with the targeted SCs prior to microextraction. Recovery was determined by evaluating the absolute peak area ratio between the two sets. Furthermore, to account for potential variations resulting from analysing different sources, the relative standard deviation (RSD) values were calculated. An RSD value of ≤ 20.0 % was deemed acceptable.

## Results

### Sorbent selection

**Fig. 2:**
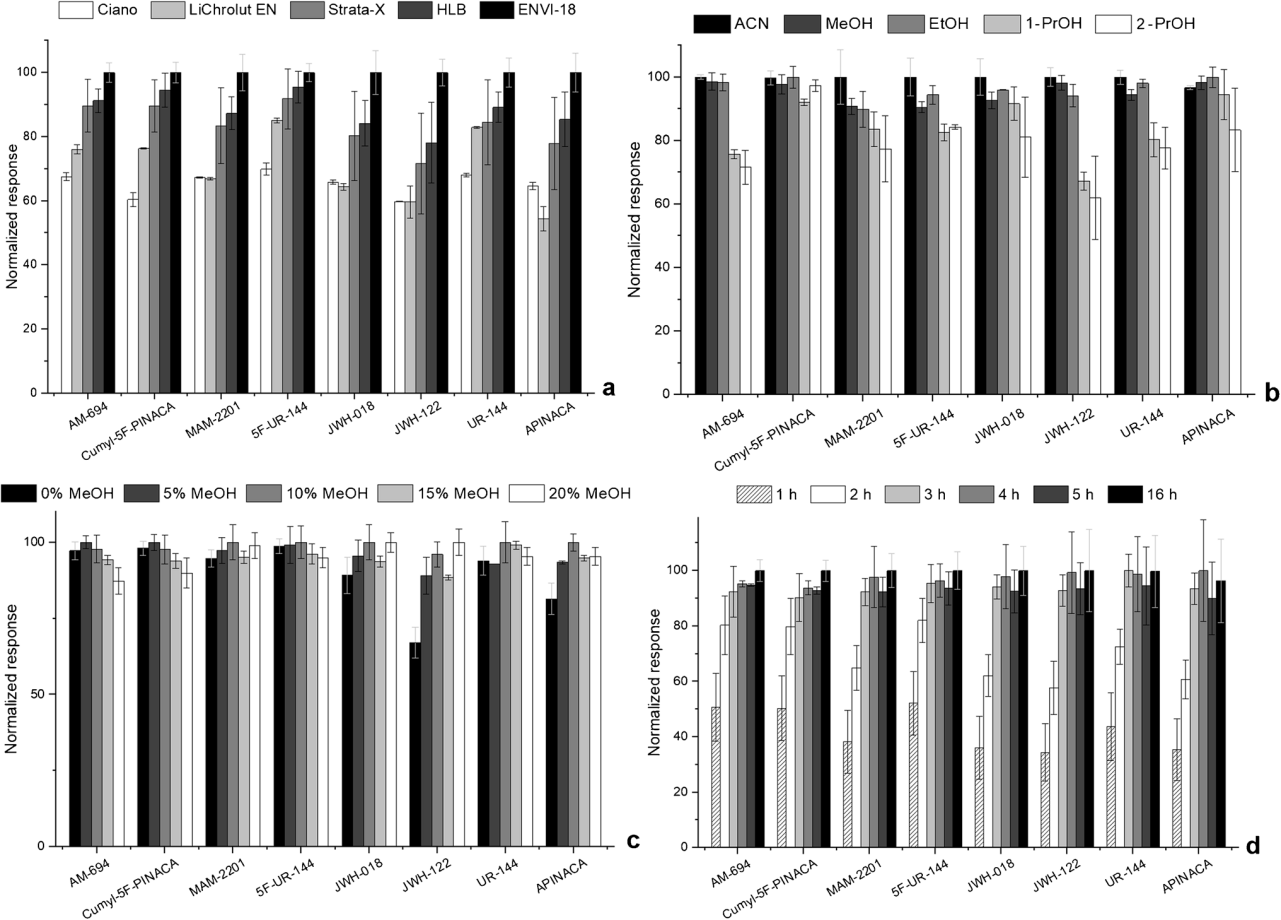
Effect of polymeric sorbent selectivity (**a**), µLD solvent (**b**), the addition of organic modifier (**c**) and equilibrium time (**d**) on the microextraction of the eight SCs in aqueous media, obtained by BAµE-µLD/HPLC-DAD methodology. The error bars represent the standard deviation of three replicates

Five polymeric phases were tested as sorbent coatings for enrichment purposes, under the following standard conditions: microextraction 3 h (1000 rpm), pH 5.5 with ultrapure water; back-extraction ACN (100 µL), 30 min under sonication treatment. The obtained results clearly demonstrate that the ENVi-18 polymer exhibited superior selectivity for all eight target compounds (Fig. 2a); hence, it was selected for further assays.

### Optimization of the microliquid desorption stage

After selecting the best sorbent phase, we decided to optimize the μLD step. Among the parameters potentially influencing this stage, we examined the solvent options (MeOH, ACN, EtOH, 1-PrOH, and 2-PrOH, as depicted in Fig. 2b) and the duration of back-extraction (5, 15, 30, 45 and 60 min, supplementary data S2). Based on the data obtained, the most favourable outcomes were achieved by employing ACN for a period of 15 min under ultrasonic treatment.

### Optimization of the microextraction stage

Furthermore, we evaluated various characteristics of the matrix, including pH, ionic strength and polarity that could influence the extraction yields. The impact of different pH values (2.0, 5.5, 8.0 and 11.0) on the microextraction efficiencies of the compounds under investigation was assessed, and it was concluded that the pH did not significantly affect the process (supplementary data S3). Additionally, the polarity of the matrix was analysed by employing an organic solvent [38], specifically MeOH (ranging from 0 to 20 % v/v). Generally, the data obtained indicated an increase in average recovery of up to 10 % MeOH, but no significant advantages were observed when employing higher concentrations (Fig. 2c). Lastly, the impact of ionic strength was investigated. This is accomplished by adding a strong electrolyte to the aqueous phase [39]. To achieve this, we added NaCl at concentrations ranging from 0 to 20% (w/v) into the matrix, and a reduction in the response of the methodology was observed (supplementary data S4).

Moreover, we examined the parameters that influence the kinetics of the microextraction process, including equilibrium time and stirring speed. The stirring speed can affect the mass transfer of analytes to the sorbent phase and accelerate the equilibrium process. Thus, we tested stirring rates of 750, 1000 and 1250 rpm, avoiding higher speeds to prevent excessive turbulence, which may compromise the stability of the device and hinder the microextraction process [31]. The data clearly indicates that a stirring speed of 1000 rpm yields the highest response (supplementary data S5). Equilibrium time was found to significantly influence the microextraction process, as it governs the kinetics of analyte interactions between the sorbent phase and the matrix [32]. Accordingly, we investigated multiple equilibrium times (1, 2, 3, 4, 5 and 16 h), and the results are illustrated in Fig. 2d. Based on the obtained data, it was determined that the microextraction kinetics are rapid, and 3 h provides sufficient time to achieve equilibrium, thereby maximizing the recovery yields of the eight target compounds from aqueous media.

In short, the optimized BAµE(ENVI-18)-µLD methodology conditions were as follows: microextraction stage 3 h (1000 rpm), pH 5.5 and MeOH 10 % (v/v); back-extraction ACN (100 μL) under 15 min of sonication.

### Method validation

The developed analytical approach allowed to obtain a linear range between 20.0 and 2000.0 µg L^−1^ for all eight SCs under study. This resulted in *r*^2^ between 0.9969 (APINACA) and 0.9995 (cumyl-5F-PINACA), with RR ≤ 14.9 % (APINACA), for all compounds, and no carryover effects were observed. This was performed by analysing a blank sample after injecting the highest standard of the calibration plot, showing any peaks of the target analytes.

The sensitivity of the methodology was checked through the LODs and LOQs calculated with S/N of 3/1 and 10/1, respectively. The developed methodology allowed to obtain LODs of 2.0 µg L^−1^ and LOQs of 10.0 µg L^−1^ for cumly-5F-PINACA, 5F-UR-144 and UR-144. For the remaining SCs, AM-694, MAM-2201, JWH-018, JWH-122 and APINACA, the LODs and LOQs were 5.0 µg L^−1^ and 15.0 µg L^−1^, respectively.

Table 1 summarizes the methodology intraday and interday accuracies and precisions, showing suitable data (bias and RSD ≤ 15.0 %). Recovery was checked in sextuplicate using two spiking levels (50.0 and 300.0 µg L^−1^). The proposed methodology (Table 1) showed excellent average recovery yields (83.4–100.5%) and the lack of significant variation using several matrices (RSD ≤ 13.3 %). To assess selectivity, ten oral fluid samples collected from drug-free individuals were tested using the proposed methodology. The results achieved were negative for all eight SCs, indicating that the method can exclude the interference from endogenous materials.

**Table 1:** Intraday and interday accuracies and precisions, as well as recovery yields, for the eight SCs in oral fluids by BAµE-µLD/HPLC-DAD methodology, under optimized experimental conditions

SCs	Spiking level (µg L^−1^)	Intraday		Interday		Recovery (± RSD, %)
		Accuracy (Bias, %)	Precision (RSD, %)	Accuracy (Bias, %)	Precision (RSD, %)	
AM-694	50.0	−1.4	7.0	−0.9	6.0	95.6 ± 4.7
	300.0	−5.5	6.2	−3.9	5.2	97.8 ± 1.7
	1000.0	10.1	4.7	6.3	4.8	
Cumyl-5F-PINACA	50.0	13.0	5.0	13.6	11.6	96.0 ± 4.6
	300.0	3.0	6.7	1.9	6.0	99.2 ± 2.4
	1000.0	8.4	4.8	9.7	5.6	
MAM-2201	50.0	−14.5	13.0	−8.0	11.2	97.4 ± 4.7
	300.0	−13.8	9.0	−9.3	8.0	99.5 ± 6.0
	1000.0	8.7	8.0	9.0	7.7	
5F-UR-144	50.0	−10.6	9.0	−5.0	7.9	98.1 ± 4.8
	300.0	−10.0	6.6	−7.4	6.3	100.5 ± 3.9
	1000.0	11.2	4.7	9.0	4.1	
JWH-018	50.0	−12.2	14.9	−5.7	11.3	91.9 ± 9.0
	300.0	−9.5	12.4	−1.4	11.0	92.4 ± 9.3
	1000.0	9.1	10.8	4.4	9.1	
JWH-122	50.0	−14.7	11.3	−14.4	9.2	91.4 ± 8.4
	300.0	−12.1	13.6	10.4	8.0	88.4 ± 12.4
	1000.0	−7.7	14.4	−1.8	8.0	
UR-144	50.0	−12.4	12.0	−11.3	8.0	94.4 ± 7.6
	300.0	14.4	12.6	−11.6	8.7	95.2 ± 8.1
	1000.0	6.7	10.5	2.0	8.8	
APINACA	50.0	−12.7	8.3	−9.9	5.7	87.9 ± 13.3
	300.0	11.9	4.8	12.0	11.0	92.7 ± 13.2
	1000.0	−12.4	14.4	−5.3	11.0	

## Discussion

SCs represent the most frequently seized new psychoactive substances (NPS) in Europe and are among the highly abused drugs worldwide, following the ‘classic’ substances [5]. Consequently, it is imperative to develop novel and environmentally friendly analytical methodologies to monitor SCs in biological samples. Oral fluid serves as a reliable matrix for short-term SC consumption monitoring due to its ease of collection and accurate results [6]. This is particularly critical for evaluating drug-impaired drivers and ensuring occupational safety. Unlike traditional drugs, which can be conveniently detected using chemical spot tests *on-site*, NPS, especially the recently introduced ones, lack such straightforward monitoring approaches. Therefore, there is an increasing demand for faster and cost-effective screening methods employing commonly available equipment in analytical toxicology laboratories.

In the present contribution, the optimized microextraction stage, using an octadecyl polymeric phase as coating material for BAμE devices, showed great performance for trace analysis of all the target compounds in oral fluid samples. This favourable outcome can be attributed to the presence of octadecyl groups in the coating phase, which likely facilitates reversed-phase mechanisms through strong hydrophobic interactions with the aliphatic groups in the SCs’ molecular structure (Fig. 1). Furthermore, the non-polar nature of the target SCs (4.74 < log *P* < 7.03 [23]) favours reverse-phase type interactions. The effectiveness of the organic modifier assays can also be explained by the inhibitory effect of MeOH on analyte adsorption to the sampling flask’s glass walls (‘wall effect’) [39], increasing their solubility, and therefore, favouring reverse-phase interactions. The hydrophobic nature of the target compounds may also explain the decreasing efficiency depicted in supplementary data S2 when higher sonication times are used. Initially, the sonication treatment facilitates rapid and complete back-extraction of the target compounds, followed by an equilibrium process where redistribution to the sorbent phase occurs. Moreover, the extraction efficiencies remain consistent across different sample pH values, as expected, since the target SCs do not ionize under the tested pH conditions [23].

**Fig. 3:**
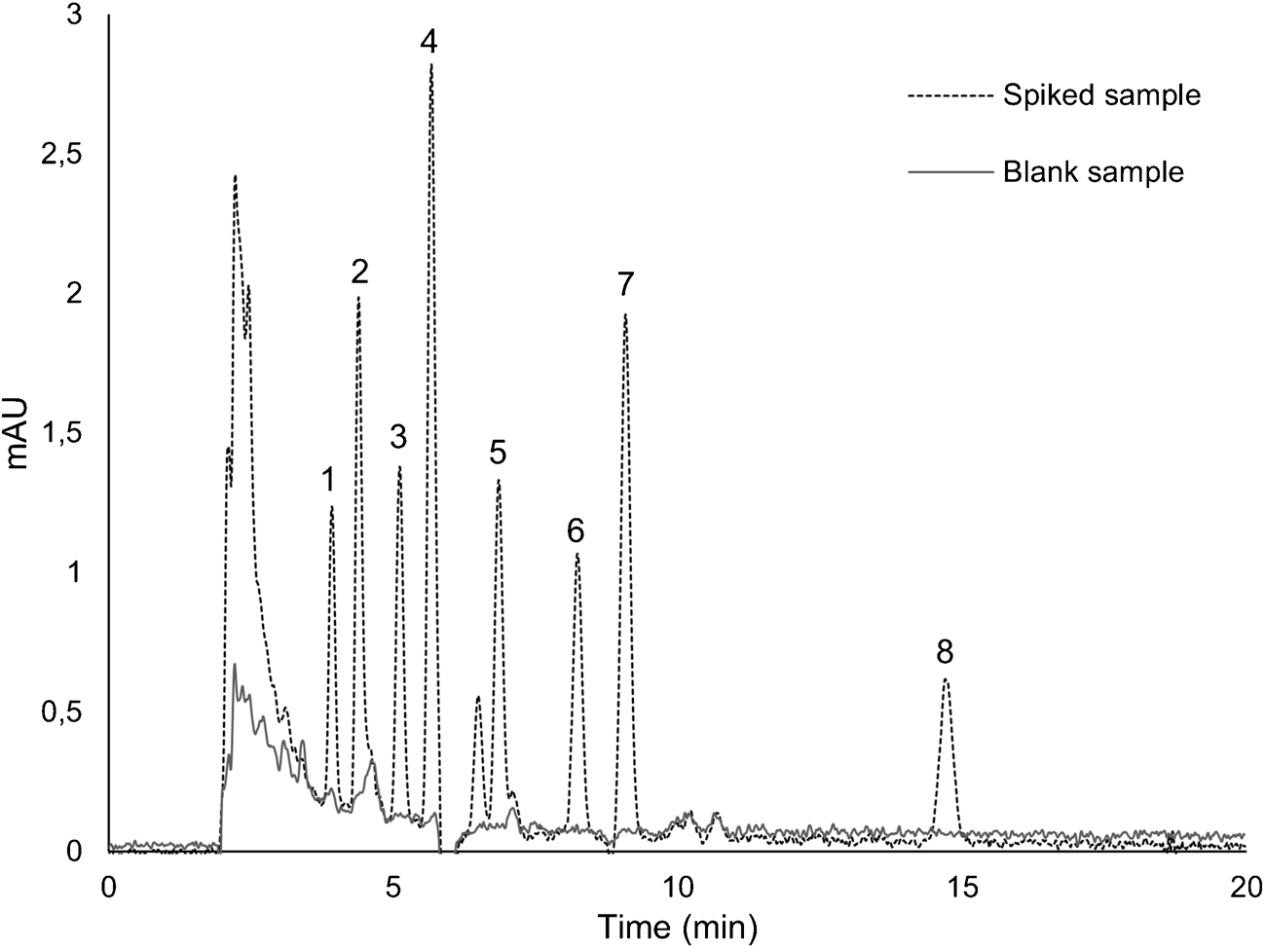
Chromatogram profiles from assays performed on spiked (50 µg L^−1^) and unspiked oral fluid samples, obtained by BAµE-µLD/HPLC-DAD methodology, under optimized experimental conditions. 1, AM-694; 2, cumyl-5F-PINACA; 3, MAM-2201; 4, 5F-UR-144; 5, JWH-018; 6, JWH-122; 7, UR-144; 8, APINACA

Several works are present in the literature regarding the analysis of SCs in oral fluids using gas chromatography-mass spectrometry (GC-MS), ultra-high-performance liquid chromatography (UHPLC) or LC-MS/MS instrumental systems [7–13, 40, 41]. It must be noted that some of these applications incorporated a sample enrichment step (e.g. LLE [11] or SPE [7, 9]) prior to instrumental analysis, which do not take into account the green analytical chemistry principles. In light of this, we propose an alternative methodology based on passive-based microextraction technology for the enrichment of eight SCs in oral fluids, leading to a reduction of organic solvent usage by 2.3 to 3.7 times compared with previous approaches [7, 9, 11]. Furthermore, our methodology is user-friendly, reducing analytical steps and eliminating time-consuming processes such as evaporation and solvent switching, in particular when compared with more classic sample preparation approaches (e.g. LLE [9] or SPE [7, 9]). When compared with miniatured enrichment techniques, Table 2, such as microextraction by packed sorbent (MEPS) or dispersive liquid-liquid microextraction (DLLME), the proposed employs less analytical steps, without compromising solvent consumption or extraction efficiencies [40, 41]. It is also worth emphasizing that the proposed technique presents suitable recovery yields when compared with other published works [7, 9–11]. Even when compared with other work that employ more sensitive and selective instrumental system (UHPLC-MS/MS [13] or GC-MS [42]), the proposed methodology exhibits similar sensitivity. Figure 3 illustrates the suitable selectivity of the developed methodology, depicting the absence of endogenous interfering peaks at the retention times of the eight SCs studied. Nevertheless, we recommend investigating exogenous compounds that might interfere with the method’s selectivity, as reported by several authors [7, 9–13].

**Table 2:** Comparison of relevant parameters between the proposed method and other published works. This assessment includes

Sample amount (mL)	Solvent usage (mL)^1^	Number of analytical steps	Recovery (%)	LOD (ng mL^−1^)	Methodology	Ref.
1	2.99	10	96–122	0.5^2^	SPE/LC-MS/MS	[7]
0.333	2.55	10	14–86	0.015–0.900	LLE/LC-MS/MS	[8]
0.5	5.035	9	56–106	0.025–1.0	SPE/LC-MS/MS	[9]
0.2	0.65	5	69–80	0.02–0.4	PP/LC-MS/MS	[10]
1	5	5	65–87	0.1–0.5	LLE/LC-MS/MS	[11]
0.1	0.1	4	68–78	0.1	PP/LC-MS/MS	[12]
0.25	0	2	100	1–20	DS/UHPLC-MS/MS	[13]
0.09	1.59	9	31–96	0.009–0.850	PP-MEPS/UHPLC-MS/MS	[40]
0.5	1.14	9	73–101	0.002–0.021	PP-DLLME/LC-MS/MS	[41]
1	0	4	n.a.	1–10	PP-SPME/GC-MS	[42]
0.5	1.35	7	88–101	2.0	PP-BAµE/HPLC-DAD	This work

*BAµE* bar adsorptive microextraction, *DLLME* dispersive liquid-liquid microextraction, *DS* dilute and shoot, *HPLC-DAD* high-performance liquid chromatography with diode array detection, *LC-MS/MS* liquid chromatography coupled to tandem mass spectrometry, *LLE* liquid-liquid extraction, *MEPS* microextraction by packed sorbent, *PP* protein precipitation, *SPE* solid-phase extraction, *SPME* solid-phase microextraction, *UHPLC-MS/MS* ultra high-performance liquid chromatography coupled to tandem mass spectrometry

*n.a.* not available

^1^During sample preparation

^2^LOQ value, LOD n.a.

Although the developed methodology was fully validated for the linear dynamic range of 20.0–2,000.0 µg L^−1^, no SCs (< LOD) were detected in the analysed matrices (10 oral fluid samples). Nonetheless, this approach remains well-suited for the analysis of such compounds in oral fluids, considering previous detections of concentrations reaching up to 35.0 and 381.0 µg L^−1^ for JWH-018 and JWH-122, respectively [7, 8]. Even so, we suggest the use of *tandem* systems (e.g. LC-MS/MS) for further studies, once an increase of selectivity and sensibility should be expected. Nonetheless, using our proposed sample preparation step, the obtained liquid extract can be injected in much faster and readily available screening instrumental system (e.g. HPLC-DAD) and be further analysed in a more advanced apparatus (e.g. LC-MS/MS) for SC confirmation. This streamlined approach significantly reduces the number of analytical steps and the overall operating time involved.

## Conclusions

The analytical methodology proposed in this study has been thoroughly optimized and validated for the simultaneous monitoring of eight SCs in oral fluid matrices. The implemented procedure demonstrated favourable analytical performance under the optimized experimental conditions, encompassing recovery, matrix effects, precision, accuracy, selectivity, sensitivity and linear dynamic ranges. However, certain limitations are present, primarily concerning sensitivity and selectivity, which can be effectively addressed by integrating the optimized microextraction-based approach with tandem systems, such LC-MS/MS.

## Supplementary Information

Below is the link to the electronic supplementary material.

Supplementary file1 (DOCX 477 KB)
