# Supplementary material for: Bar adsorptive microextraction and liquid chromatography-diode array detection of synthetic cannabinoids in oral fluid
Source: Anal Bioanal Chem. 2024 Sep 11;416(28):6307–16. doi: 10.1007/s00216-024-05517-0 (PMC11541396; doi:10.1007/s00216-024-05517-0)
Supplement: Supplementary file 1 — Supplementary file1 (DOCX 477 KB) [file 216_2024_5517_MOESM1_ESM.docx]

**Supplementary material**


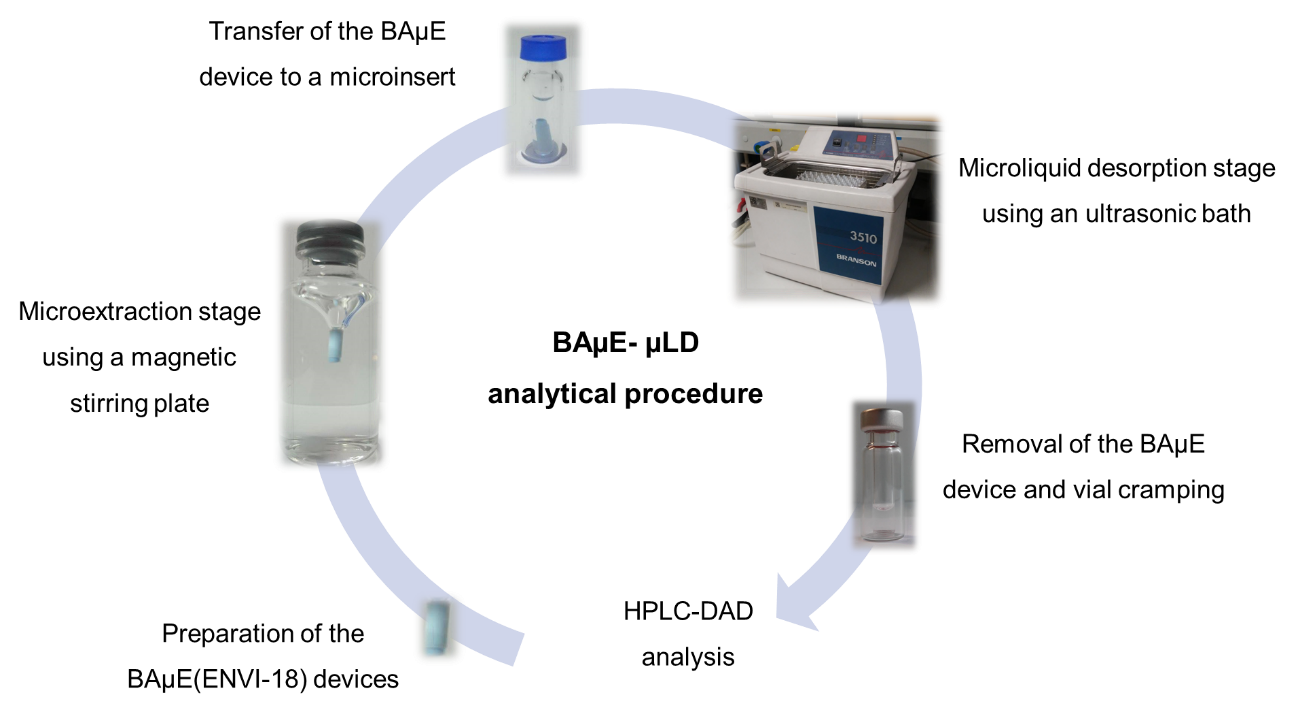


**Supplementary data S1** - Simplified schematic of the proposed experimental procedure.


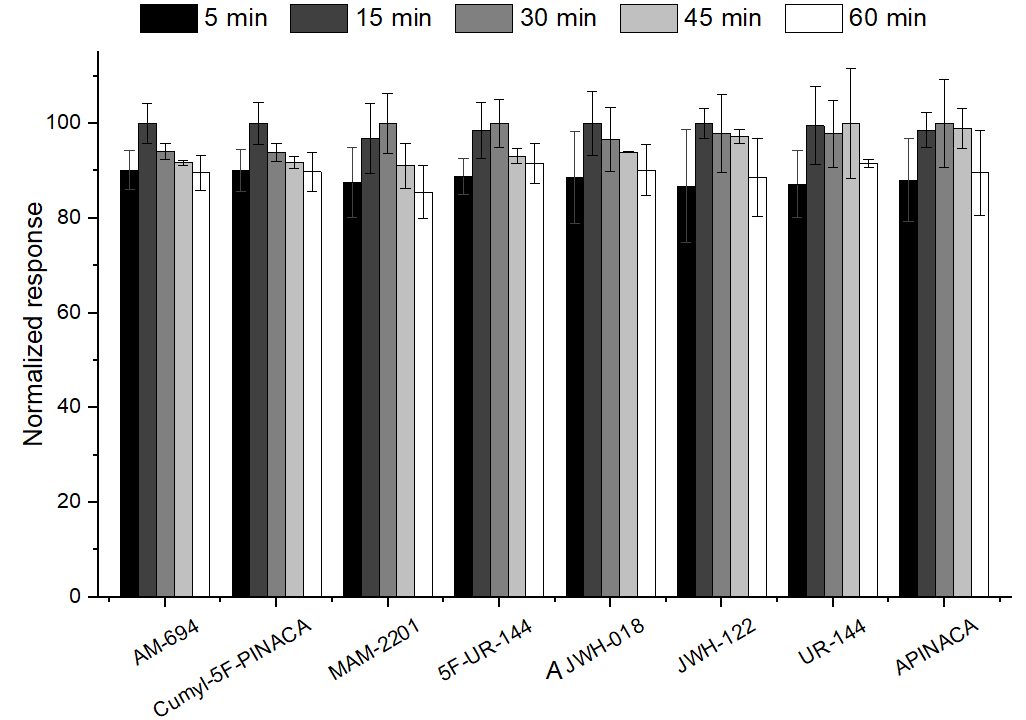


**Supplementary data S2** - Effect of sonication time on the back-extraction process for the microextraction of the eight SCs in aqueous media obtained by BAµE-µLD/HPLC-DAD methodology. The error bars represent the standard deviation of three replicates.


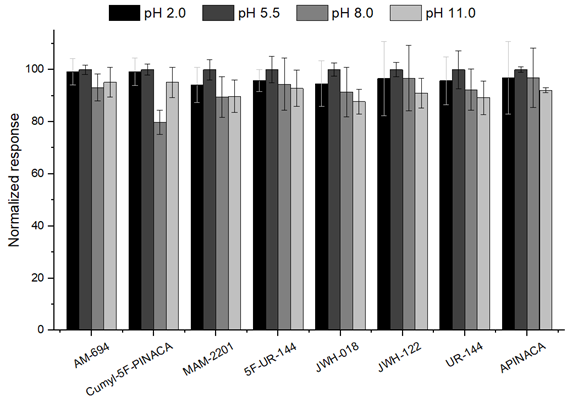


**Supplementary data S3 -** Effect of matrix pH on the microextraction of the eight SCs in aqueous media obtained by BAµE-µLD/HPLC-DAD methodology. The error bars represent the standard deviation of three replicates.


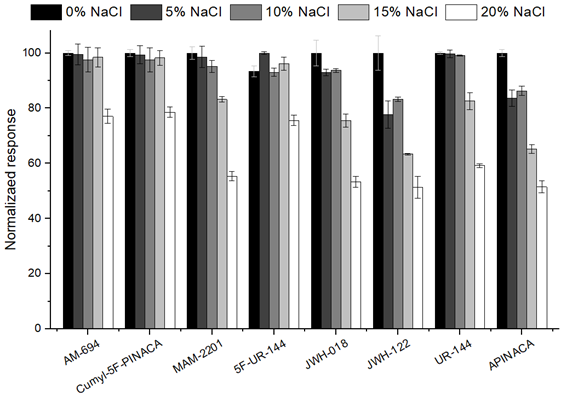


**Supplementary data S4** - Effect of ionic strength on the microextraction of the eight SCs in aqueous media obtained by BAµE-µLD/HPLC-DAD methodology. The error bars represent the standard deviation of three replicates.


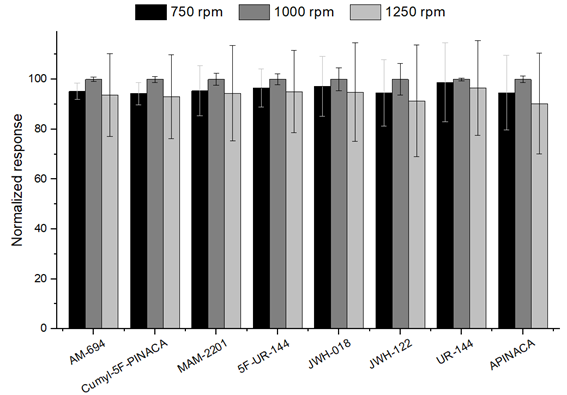


**Supplementary data S5** - Effect of stirring rate on the microextraction of the eight SCs in aqueous media obtained by BAµE-µLD/HPLC-DAD methodology. The error bars represent the standard deviation of three replicates.
